# *Streptococcus pneumoniae* Carriage Prevalence in Nepal: Evaluation of a Method for Delayed Transport of Samples from Remote Regions and Implications for Vaccine Implementation

**DOI:** 10.1371/journal.pone.0098739

**Published:** 2014-06-06

**Authors:** Sarah Hanieh, Mainga Hamaluba, Dominic F. Kelly, Jane A. Metz, Kelly L. Wyres, Roberta Fisher, Rahul Pradhan, Disuja Shakya, Lochan Shrestha, Amrita Shrestha, Anip Joshi, Jocelyn Habens, Bishnu D. Maharjan, Stephen Thorson, Erik Bohler, Ly-Mee Yu, Sarah Kelly, Emma Plested, Tessa John, Anja M. Werno, Neelam Adhikari, David R. Murdoch, Angela B. Brueggemann, Andrew J. Pollard

**Affiliations:** 1 Oxford Vaccine Group, Department of Paediatrics, University of Oxford, and the NIHR Oxford Biomedical Research Centre, Oxford, United Kingdom; 2 Patan Academy Paediatric Research Unit, Patan Academy of Health Sciences, Kathmandu, Nepal; 3 Department of Zoology, University of Oxford, Oxford, United Kingdom; 4 Okhaldhunga Community Hospital, Okhaldhunga, Nepal; 5 Nuffield Department of Primary Care and Health Sciences, University of Oxford, Oxford, United Kingdom; 6 Department of Pathology, University of Otago, Christchurch, New Zealand; 7 Canterbury Health Laboratories, Christchurch, New Zealand; Centers for Disease Control & Prevention, United States of America

## Abstract

**Background:**

Pneumococcal disease is a significant cause of morbidity and mortality in young children in Nepal, and currently available pneumococcal conjugate vaccines offer moderate coverage of invasive disease isolates.

**Methods:**

A prevalence study of children aged 1.5 to 24 months in urban and rural Nepal was conducted. In the urban group, nasopharyngeal swabs (NPS) were transported using silica desiccant packages (SDP) with delayed processing (2 weeks), or skim-milk-tryptone-glucose-glycerin (STGG) with immediate processing (within 8 hours). Pneumococcal nasopharyngeal carriage prevalence, serogroup/type distribution and isolate genotypes (as defined by multilocus sequence typing) were determined.

**Results:**

1101 children were enrolled into the study: 574 in the urban group and 527 in the rural group. Overall carriage prevalence based on culture from specimens transported and stored in STGG was 58.7% (337/574), compared to 40.9% (235/574) in SDP. There was concordance of detection of pneumococcus in 67% of samples. Using the SDP method, pneumococcal carriage prevalence was higher in the rural population (69.2%; 364/526) compared to the urban population (40.9%; 235/574). Serogroup/type distribution varied with geographical location. Over half of the genotypes identified in both the urban and rural pneumococcal populations were novel.

**Conclusion:**

The combination of delayed culture and transport using SDP underestimates the prevalence of pneumococcal carriage; however, in remote areas, this method could still provide a useful estimate of carriage prevalence and serogroup/type distribution. Vaccine impact is unpredictable in a setting with novel genotypes and limited serotype coverage as described here. Consequently, continued surveillance of pneumococcal isolates from carriage and disease in Nepali children following the planned introduction of pneumococcal conjugate vaccines introduction will be essential.

## Introduction

*S. pneumoniae* (the ‘pneumococcus’) plays a major role in early childhood morbidity and mortality and results in the death of up to 1 million children annually throughout the world [1]. There is a significant burden of pneumonia and pneumococcal disease among children presenting to hospitals in Kathmandu, Nepal [2]–[4].

Pneumococcal protein-polysaccharide conjugate vaccines are efficacious against invasive pneumococcal disease and pneumonia from early infancy [5]–[7]. Several global initiatives are driving the introduction of these vaccines into the immunization schedules of the poorer countries of the world where the disease burden is highest. Nepal is one of the countries eligible to apply for GAVI (Global Alliance on Vaccines and Immunization) [8] funding for introduction of the pneumococcal vaccine into the routine immunization schedule, with introduction currently set for late 2014.

Prior to introducing a vaccine, reliable serogroup/type information is required from circulating disease-causing strains in order to ensure that protection is conferred against the prevalent regional serogroups/types and to enable the monitoring of prevalence changes after vaccine introduction. Preliminary serotype distribution data from Nepal indicated that just over 50% of invasive pneumococcal disease could be prevented with a 10- (serotypes 1, 4, 5, 6B, 7F, 9V, 14, 18C, 19F, and 23F) or 13-valent vaccine (serotypes 1, 3, 4, 5, 6A, 6B, 7F, 9V, 14, 18C, 19A, 19F, 23F [2]. Evaluation of the pneumococcal population genetic structure prior to conjugate vaccine introduction also allows for investigation of changes that occur post-vaccine introduction [9].

It is challenging to transport and process nasopharyngeal swabs (NPS) from remote areas. In 2003 a World Health Organization (WHO) working group published a recommended method using skim-milk-tryptone-glucose-glycerin (STGG) for the collection and processing of NPS [10]. Logistically this method is not feasible for remote areas with little access to microbiological facilities. An alternative method, silica desiccant packages (SDP), has previously been shown to allow successful transport and storage of pneumococci for up to 25 days [11], but there is no data to confirm comparable performance to STGG across different serotypes.

In this study we collected pneumococci from the nasopharynx of healthy children <2 years of age living in either an urban or a rural setting in Nepal, prior to introduction of the pneumococcal vaccine. The objectives of the study were to assess: i) the concordance between STGG vs SDP transport in the recovery of pneumococci in an urban setting; ii) the carriage prevalence and serogroup/type distribution in an urban vs rural setting; and iii) the genotypes circulating in the pneumococcal population prior to vaccine introduction. Isolates were genotyped by multilocus sequence typing (MLST).

## Methods

### Ethics Statement

The present study was conducted according to the principles expressed in the Declaration of Helsinki. Written informed consent was obtained from parents/guardians of the children, after a full explanation of the purpose, nature and risk of all procedures used. The protocol and consent form were approved by the Oxford Tropical Research Ethics Committee (Reference number 5908) and the Nepal Health Research Council.

### Design overview

This was a prevalence study of children residing in rural and urban Nepal. The urban study took place from January to September 2009 in Patan Hospital, Kathmandu, and the rural study took place from October to December 2009, in Okhaldhunga Hospital, Nepal.

### Setting

Patan is a sub-metropolitan city of Nepal (population ∼163,000). Patan Hospital provides care to residents of Kathmandu and surrounding valley regions [12], and the ‘under-fourteen’ outpatient clinic serves as a primary health care facility for local children. Okhaldhunga Hospital is a small community hospital that serves four surrounding districts (population >250,000), and is situated in a remote region of eastern Nepal.

### Study Participants

Study participants were recruited in equal numbers from each of three age groups: 1.5–6 months; >6 to 12 months; and >12–24 months. In both settings, parents and guardians of healthy children attending the outpatient clinic for vaccination or routine check-up, or who were visiting other relatives in hospital, were approached regarding recruitment of their children into the study. In Okhaldhunga, parents were also approached in the Okhaldhunga Bazaar, and in health camps. Exclusion criteria included the presence of a febrile illness/axillary temperature >38°C, or documentation of previous pneumococcal vaccination. Information on demographics and risk factors for pneumococcal carriage was collected, and a NPS was taken by trained staff.

### Pneumococcal isolate collection, transport and processing

For children recruited to the urban arm of the study at Patan Hospital, rayon NPS were manually doubly intertwined and used to swab the nasopharynx of participating children according to WHO standard procedures [10]. After sampling, the two NPS were aseptically separated and one swab was put into a tube of 0.5–1 ml of STGG transport medium, the other into a sterile SDP (Gee Jay Chemicals). The STGG swab was transported on ice to the laboratory and plated within 8 hours of collection [10]. The SDP swab was transported to the laboratory within four hours, although microbiological processing was done after fourteen days, in order to match the timing of the SDP swabs from Okhaldhunga.

In Okhaldhunga, a single NPS was taken from the nasopharynx of study participants by trained study nurses or physicians using the methods described above. The swab was put into a sterile SDP. The SDP swab was maintained at ambient temperature and transported to Patan Hospital laboratory where microbiological culture was done 14 days after the swab was taken.

### Microbiology

All swab specimens were cultured onto Columbia agar plates containing 5% sheep blood, and incubated overnight at 35–37°C plus 5% CO_2_. A single colony of pneumococcus was picked for confirmation and serotyping. Identification of pneumococci was based on colony morphology, optochin susceptibility and/or bile solubility. Swabs were processed and cultured in Patan Hospital microbiology laboratory, and isolates were sent to the University of Oxford, UK for serogrouping/typing and genotyping.

### Serogrouping/typing and genotyping

Isolates were serogrouped/typed by PCR amplification using published methods [13]–[19]. Serogroups/types were determined using a range of 42 different PCR primers (Table S1). Standard PCR amplifications were performed and amplicons were visualized in an agarose gel. Control *cpsA* primers were used in every reaction. Pneumococci that were optochin-susceptible but *cpsA*-negative were considered to be nontypeable. A subset of pneumococci were also tested using the Quellung reaction: isolates that were *cpsA*-negative and thus putatively nontypeable (n = 66); and isolates that were PCR-positive for either serotype 14 (n = 40) or 15B/C (n = 54), but where there was the potential for cross-reaction among the PCR primers.

Patan STGG isolates (n = 299) and Okhaldhunga isolates (n = 300) were genotyped by MLST using standard protocols (Table S2) [18], [19]. Frequent PCR failures using standard *gdh* MLST primers resulted in a redesign of *gdh* primers for this study. Alleles and STs were assigned using the MLST database (http://spneumoniae.mlst.net/) and clonal complexes (CCs), clusters of genotypes descended from a recent common ancestor, were defined using goeBURST and displayed using Phyloviz [20], [21], The genetic diversity of the isolates found in Kathmandu and Okhaldhunga was compared by calculating the Simpson index of diversity for each population, [22] and 95% confidence intervals were calculated using the method of Grundmann [23].

### Statistical Analyses

Data were analyzed using STATA Version 11 (StataCorp, College Station, TX, USA). Categorical data are presented as percentages with frequency. Carriage prevalence estimates and 95% confidence intervals were calculated using the Binomial Exact Method. Comparison of carriage prevalence between the urban and rural regions was performed using the chi-squared test.

## Results

1,101 children were enrolled in the study from January to December 2009: 574 children from urban Kathmandu and 527 from rural Okhaldhunga. The demographics were similar between the two groups except that households in Okhaldhunga had a higher mean number of residents compared to Kathmandu (5.5 vs 4.9; *P*<0.001) (Table 1). Nasopharyngeal swab results were available for 574 participants in the urban group and 526 participants in the rural group (one swab was lost during transport). Results of serotyping confirmation by Quellung are summarized in Table S3.

**Table 1:** Baseline demographic characteristics of participants.

	Total	Rural	Urban
	(N = 1100)^a^	(N = 526)^b^	(N = 574)
Characteristic	No. (%)	No. (%)	No. (%)
Male gender	583 (53.0)	264 (50.2)	319 (55.6)
Age in months			
1.5 to 6	352 (32.0)	159 (30.2)	193 (33.6)
>6 to 12	376 (34.2)	184 (35.0)	192 (33.4)
>12 to 24	372 (33.8)	183 (34.8)	189 (33.0)
Number of people living in household			
0–1	1 (0.1)	0 (0)	1 (0.2)
2–5	742 (67.4)	314 (59.7)	428 (74.6)
6–10	327 (29.7)	196 (37.3)	131 (22.8)
>10	30 (2.7)	16 (3.0)	14 (2.4)

a. N = number of children.

b. Nasopharyngeal swab missing from one child in rural cohort.

### Pneumococcal carriage using STGG transport method (urban group)

**Figure 1:**
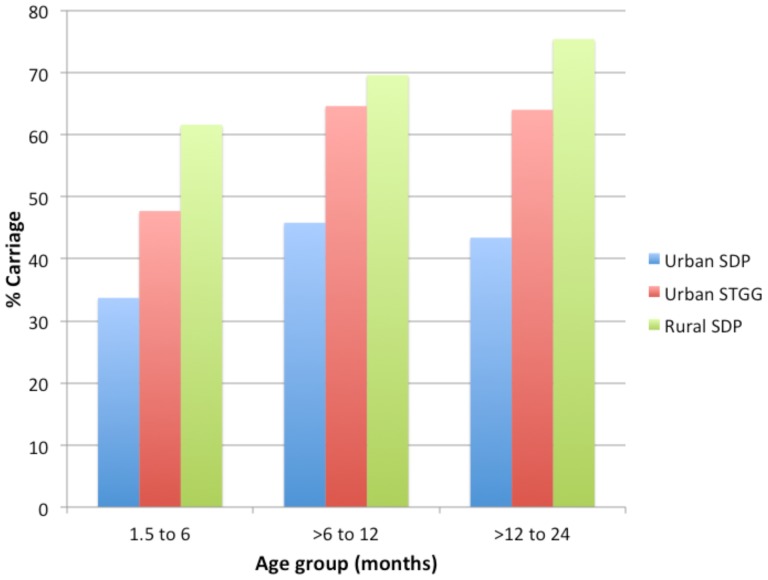
Age-specific pneumococcal carriage rates.

Carriage prevalence determined by the STGG method in Patan Hospital microbiology laboratory was 58.7% (337/574); however, following transport to the University of Oxford, UK, only 330 isolates collected using the STGG method contributed to serogroup/type data as several cultures were non-viable or not stored and therefore were unable to be typed. The highest carriage prevalence was seen in the >6 to 12 month age group (64.6%; 124/192; *P* = 0.001; Figure 1). Serogroup/type specific carriage prevalence ranged from 0.3% to 14.4%, with the twelve most common serogroups/types (6, NT, 15B/C, 34, 21, 11, 17F, 23A, 23F, 14, 19F and 35B) comprising 62.1% of all isolates.

### Comparison of STGG transport method with SDP transport method (urban group)

**Figure 2:**
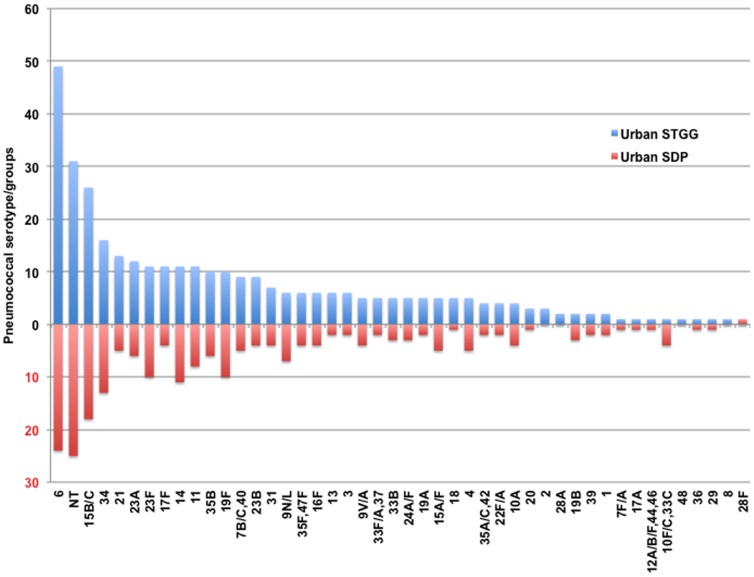
Frequency of pneumococcal serogroups/types in urban STGG samples compared to urban SDP samples.

**Figure 3:**
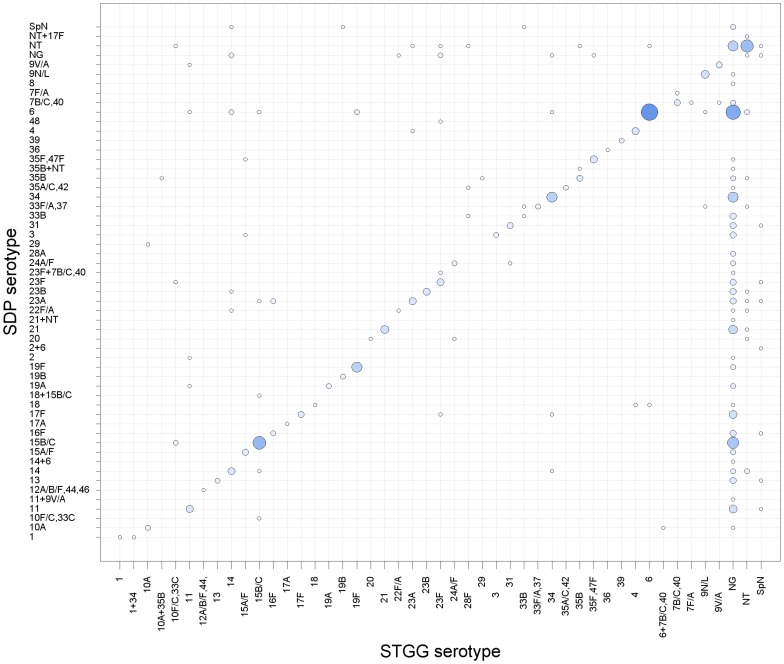
Serogroup/type concordance between the different swab transport methods. The size of data points is proportional to the frequency of isolates. Spn = *Streptococcus pneumoniae*, NT = nontypeable, NG = no growth.

Overall carriage prevalence based on culture from specimens transported and stored in STGG was 58.7% (337/574), and in SDP 40.9% (235/574) (chi-squared test P<0.001). For participants in whom any pneumococcus was detected (in STGG and/or SDP) there was concordance of detection of pneumococcus in 67% of samples. The remaining 33% were discordant with 30% being STGG +/ SDP-, and 3% being STGG -/ SDP+. The serogroup/type distribution for urban STGG versus SDP samples is presented in Figure 2. The four most prevalent STGG serogroups/types were shared with those from SDP (6, NT, 15B/C, and 34). The fourteen most prevalent STGG serogroups/types constituted 68% of isolates and twelve were shared with those from SDP. Of the two STGG serogroups/types not shared with SDP the mean difference in rank order was 11 positions (range 9–14 positions). For the 211 participants in whom pneumococci were detected by both STGG and SDP (and serogroup/types were available), discordant serogroups/types were seen in 27.5% (58/211) of individuals (Figure 3).

Among the carriage isolates identified using STGG 11.5% (39/340) were of serogroups/types which have been shown to cause disease in this population (1, 14, 23B, 23F, 8, 9V/A) [3]. These were found at similar frequencies using both methods (Figure 2).

### Comparison of urban with rural pneumococcal carriage using SDP transport method

**Figure 4:**
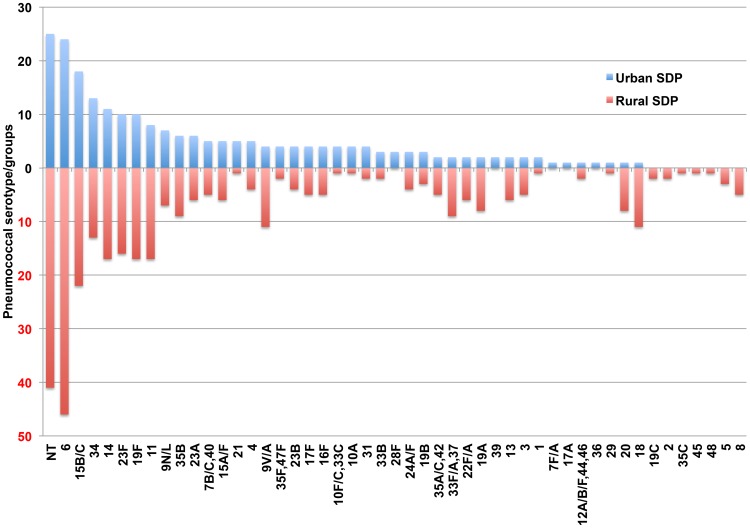
Frequency of pneumococcal serogroups/types in urban SDP compared to rural SDP samples.

Carriage was significantly higher in the rural population compared to the urban population (69.2% vs 40.9%; *P*<0.001; using SDP) and was highest in the >12 to 24 month age group (75.4%, 138/183; *P* = 0.03; Figure 1). Following transport to the University of Oxford, UK, only 350 isolates collected using the SDP method in the rural area contributed to serogroup/type data as several cultures were non-viable and therefore were unable to be typed. The distribution of serogroups/types from SDP in urban versus rural samples is presented in Figure 4. The five most prevalent rural serotypes constituted 41% of total isolates and three were shared with the urban group (6, NT and 15B/C). The fifteen most prevalent rural serotypes constituted 72% of isolates and ten were also in the top fifteen for the urban group. Of the remaining five serogroups/types not shared with urban group the mean difference in serogroup/type rank between rural and urban distributions was 21 positions (range 13–28 positions).

### Genotyping

All isolates recovered from STGG swabs from Kathmandu and all isolates from SDP samples from Okhaldhunga were sent for genotyping. The number of isolates genotyped in total was 599: 299 isolates from Kathmandu and 300 from Okhaldhunga. Isolates with nonviable cultures were unavailable for genotyping. Fourteen isolates (Kathmandu, n = 8; Okhaldhunga, n = 6) had incomplete ST profiles due to repeated PCR failures at 1 or 2 loci even with redesigned primers, presumably due to divergent sequence in the primer binding regions. Two serotype 19F isolates, one from each location, were missing *ddl*, but were identical at 6 of 7 loci to ST7663^19F^ (note: ST serogroup/type). Three serotype 34 isolates from Okhaldhunga were missing *xpt*, but were identical at all other loci to ST7570. The remaining nine isolates had different allelic profiles. These 14 isolates were excluded from further analyses.

307 unique STs were detected among 585 fully genotyped isolates. 190 different STs were found in Kathmandu, 162 different STs were found in Okhaldhunga, and isolate representatives of 44 STs were found in both locations. 232 STs were newly recognised as a result of this study and these novel STs described 59% (n = 347) of the total collection of isolates. The genetic diversity (D) estimates for the isolates found in each location were not significantly different: Kathmandu, 99.2% (95% CI, 98.9–99.5%); and Okhaldhunga, 99.0% (95% CI, 98.7–99.3%).

**Figure 5:**
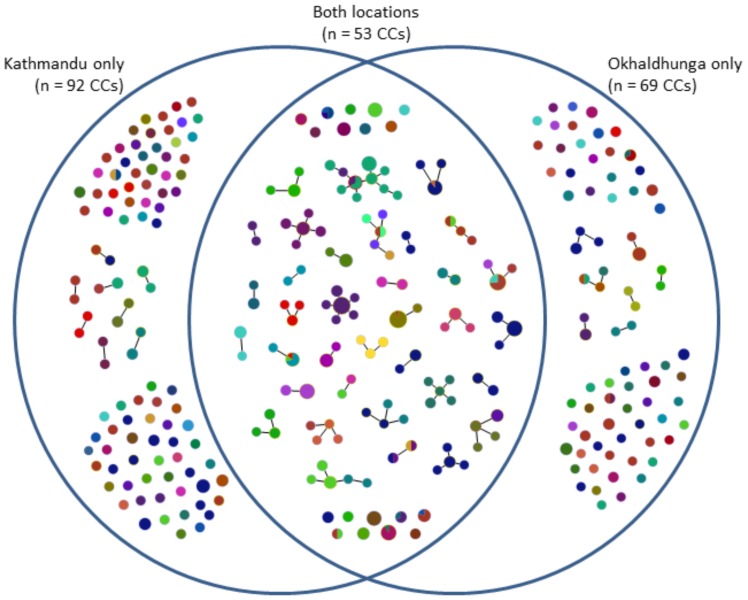
Clonal complexes (CCs) found in only one geographical location, or in both locations. Colors indicate different serogroups/types. The size of each circle is drawn in proportion to the number of isolates within that CC.

The 307 unique STs clustered into 214 CCs, which included 123 STs that had only one isolate representative (21% of the collection of 585 isolates). 92 CCs were found in Kathmandu only, 69 CCs in Okhaldunga only, and 53 CCs were comprised of pneumococci from both locations (Figure 5). Overall, there were 10 major CCs that were comprised of ≥10 isolates each and another 18 CCs with 5–9 isolates each (Table 2). Two major CCs (4560 and 4908) were comprised of a single ST, i.e. no closely-related variants were detected. Only CC63 was also an internationally-distributed CC, having been found in many countries worldwide (http://spneumoniae.mlst.net/). Isolate representatives of all the major CCs were found in both Kathmandu and Okhaldhunga, although most predominated in one of the two locations (Table 2).

**Table 2:** Clonal complexes identified in each study location.

	No. of isolates			
Clonal complex^a^	Total	KAT^b^	OKH^b^	Serogroup/type(s)
63	28	10	18	14 (n = 22); NT (n = 6)
4209	28	18	10	15B/C
6025/7595	22	7	15	11A (n = 21); 9V/A (n = 1)
8164	17	6	11	35B (n = 12); 18 (n = 4); 17F (n = 1)
5613	17	6	11	6
5281/7619	13	10	3	17F
4217	12	5	7	6 (n = 11); 39 (n = 1)
ST4560_Single	12	11	1	34 (n = 11); 11B (n = 1)
5080	12	10	2	23A
ST4908_Single	10	2	8	9V/A
ST7689_Single	9	4	5	23F
1078	9	5	4	23F (n = 7); 19F (n = 2)
6332	8	1	7	13 (n = 4); 15A/F (n = 4)
7600/7591	8	7	1	21 (n = 6); 23F (n = 1); 9N/L (n = 1)
5282/7670	7	1	6	19A
ST6029_Single	7	5	2	31
7651/5392	7	1	6	20
7611/7597	7	0	7	NT
2712	6	2	4	4
6030	6	5	1	16F
5292	6	5	1	3
3735	6	3	3	10A, 10F(10C/33C) (n = 2 ea); 35B, unknown^c^ (n = 1 ea)
7585	6	5	1	6
7692/5068	5	2	3	18
ST6132_Single	5	1	4	NT (n = 4); 35A(35C/42) (n = 1)
7590	5	0	5	6
7536/7538/7562	5	1	4	33F/A, 37
ST6031_Single	5	5	0	6
Other^d^	297	153	144	—
Total	585	291	294	—

a. Clonal complexes are named with the sequence type (ST) predicted to be the ancestral ST. When the ancestral ST could not be determined, all STs in the clonal complex are indicated (e.g. 6025/7595). When an ST had no related variants it is marked as “STxx_Single”. The first 10 CCs listed were considered to be major CCs and the remainder were minor CCs.

b. KAT  =  Kathmandu; OKH  =  Okhaldhunga.

c. One serotype was unknown because the isolate was PCR-negative for the serotyping primers and the culture was nonviable (and thus unavailable for serotyping by Quellung).

d. Clonal complexes with: 4 isolates each, n = 15; 3 isolates each, n = 18; 2 isolates each, n = 30; and 1 isolate each, n = 123.

There was a strong association between serogroup/type and CC. Eight of the ten major CCs and 14 of the 18 minor CCs were comprised of isolates of either a single, or clearly predominant (only a single isolate variant), serogroup/type (Table 2 and Figure 5). In none of these cases did the minority serogroup/type also represent a change in ST within the CC. Overall, the serogroups/types found among the major and minor CCs were among those expected in carriage (Table 2). Globally, CC63 isolates predominantly express serotype 14 and that was true for these Nepali CC63 isolates as well.

## Discussion

This is the first study to compare pneumococcal carriage in children in urban and rural Nepal, and evaluated an SDP method to facilitate work in remote locations. Pneumococcal carriage is common in young Nepali children, with prevalence being higher in rural as compared to urban locations. Whilst the most prevalent serotypes/groups of pneumococci are shared by children in rural and urban locations, there do appear to be some differences in serogroup/type and genotype distribution.

The transport and delayed processing of pneumococcal isolates using SDP offers a major advantage for remote areas with no laboratory facilities. However, there are few data regarding the sensitivity of the SDP method compared to STGG and it is possible that different methods may bias towards detection of some serogroup/types. The only previously published study using SDP did not have an STGG comparison group [11]. In our study, although the sensitivity of SDP versus STGG was 67%, the overall serogroup/type distribution was similar between methods. Twelve of the 14 most prevalent STGG serogroups/types were common to SDP and the other 2 differed in rank order by 9–14 positions. In future studies it would be useful to assess broth enrichment pre-culture following SDP isolate storage as a method for increasing detection of pneumococci and improving sensitivity [16].

Although the overall population serogroup/type distribution was similar as assessed by the differing methods we found significant serogroup/type discordance for pairs of pneumococci isolated from STGG and SDP in the same participant. The most likely explanation is the recognised occurrence of multi-serotype carriage [24]. This phenomenon is especially important to understand in the context of the likely occurrence of serotype replacement following the introduction of routine pneumococcal immunisation.

The pneumococcal carriage prevalence in this study is broadly similar to studies in other low-income countries in Asia [25]–[27]. Pneumococcal carriage is acquired in the first few months of life and a similar prevalence then observed from 6–24 months of age. The most prevalent pneumococcal serogroups/types reported consistently in other studies in Asia include NT, 6, 14, 15, 19, 23 and 34 [28]–[30]. Of these seven prevalent Asian serogroups/types all were present in the ten most prevalent serogroups/types in this study with serogroup 6 and 15B/C isolates predominating.

The finding of higher carriage rates in children residing in rural areas is consistent with previous studies, and is likely to be due to a combination of socio-economic, environmental or genetic differences. Lower socio-economic conditions may predispose to pneumococcal carriage within this setting, with the potential for an increased risk of underlying or pre-existing medical conditions, recent viral respiratory infection, indoor air pollution and household crowding [31]–[33]. However, pneumococcal carriage rates observed in this rural population are an underestimate of true carriage prevalence due to the lower sensitivity of SDP versus STGG.

Overall there was a similar serogroup/type distribution in rural and urban settings with ten of the fifteen most prevalent serogroups/types in the rural area shared with those from the urban. The factors leading to some serotypes being more prevalent in a particular region are not well understood, but the difference in overall rural versus urban carriage prevalence is indicative of differences in transmission dynamics as discussed above. Any variation in ambient temperatures between Okhaldhunga and Kathmandu might differentially affect some serogroups/types during storage/transport. A further possible difference is season which has been shown to affect pneumococcal transmission in other settings [25].

MLST remains the gold standard for genotyping pneumococci, but until fairly recently the majority of data were generated on isolates recovered from study subjects/patients in Europe and North America and little information was available from resource-poor regions of the world. Introduction of either the 10- or 13-valent conjugate vaccines will perturb the pneumococcal population; therefore, it is likely that serotype replacement will occur, i.e. vaccine serotypes (and their associated STs) will decrease and nonvaccine serotypes (and their associated STs) may increase in prevalence, with the potential for increased nonvaccine serotype disease. Serotype switching, or the genetic exchange of the capsular locus, may also occur after mass vaccination [5]–[7], [9], [28], [34].

In Nepal we detected a large proportion of novel STs in both the urban and rural pneumococcal populations, which was not unexpected since MLST data from Nepal were limited prior to our study. Vaccine impact is unpredictable in a setting with novel genotypes and limited serotype coverage, thus it emphasises the need for continued surveillance of the pneumococcal population after conjugate vaccine introduction.

The strengths of our study are the collection of isolates from different geographic regions, from large numbers of children, as well as a design allowing comparison of STGG and SDP as transport media and concordance/discordance of serogroups/types. Monitoring of carriage and serotype distributions prior to vaccine introduction is important, as it can provide unique insight into the potential impact of vaccine on serogroup/type-specific carriage prevalence and changes in the population genetic structure after pneumococcal conjugate vaccine is introduced in 2014 in this region.

Limitations of this study include the potential for selection bias in participant recruitment, although we attempted to reduce this with recruitment from a wide variety of sites, and exclusion of any participant who had an intercurrent illness. The urban and rural studies were also carried out at different time periods of the same year, and therefore differences in pneumococcal carriage prevalence as a result of seasonal effects should be considered.

This study has demonstrated that there is a high prevalence of pneumococcal carriage in Nepal, and that the carriage rate and serogroup/type distributions vary depending on the region of the country, with the highest carriage rates found in rural areas. Isolated or remote areas are often discounted when collecting nasopharyngeal carriage data due to challenging logistics; however, it is important to consider significant variations in findings that may occur within different regions of the same country, particularly when planning public health interventions such as vaccine introduction. SDP may underestimate prevalence rates of nasopharyngeal carriage, and further studies of this transport medium and alternative transport methods are required, in order to improve the understanding and surveillance of pneumococcal carriage in populations that are difficult to investigate with optimal conventional procedures.

## Supporting Information

Table S1 Primers used for PCR serotyping of pneumococci recovered from children in Nepal.
(DOCX)

Table S2 Primers Used for Multilocus Sequence Typing.
(DOCX)

Table S3 Results of serogroup/type retesting by Quellung.
(DOCX)
