# Supplementary material for: Streptococcus pneumoniae Carriage Prevalence in Nepal: Evaluation of a Method for Delayed Transport of Samples from Remote Regions and Implications for Vaccine Implementation
Source: PLoS One. 2014 Jun 6;9(6):e98739. doi: 10.1371/journal.pone.0098739 (PMC4048273; doi:10.1371/journal.pone.0098739)
Supplement: Table S1 — Primers used for PCR serotyping of pneumococci recovered from children in Nepal. (DOCX) [file pone.0098739.s001.docx]

| **Table S1. Primers used for PCR serotyping of pneumococci recovered from children in Nepal.** | | | | | |
| --- | --- | --- | --- | --- | --- |
|  |  |  |  |  |  |
| **Serotype** | **Primer name** | **Sequence (5’ – 3’)** | **Amplicon (bp)** | **Reference** | **Comments** |
|  |  |  |  |  |  |
|  |  |  |  |  |  |
| 1 | 1-f | CTC TAT AGA ATG GAG TAT ATA AAC TAT GGT TA | 280 | 1, 2 | Blast hits to 1 |
|  | 1-r | CCA AAG AAA ATA CTA ACA TTA TCA CAA TAT TGG C |  |  |  |
|  |  |  |  |  |  |
| 2 | 2F | GTC ATT GTT ACG ATT AGT TTC GAT AGT TGA GG | 381 | 3 | Blast hits to 2 |
|  | 2R | AAT TCA ATT CCT AAG TCC TCT TCC ATA AAC TC |  |  |  |
|  |  |  |  |  |  |
| 3 | 3-f | ATG GTG TGA TTT CTC CTA GAT TGG AAA GTA G | 371 | 1, 2 | Blast hits to 3 |
|  | 3-r | CTT CTC CAA TTG CTT ACC AAG TGC AAT AAC G |  |  |  |
|  |  |  |  |  |  |
| 4 | 4-f | CTG TTA CTT GTT CTG GAC TCT CGA TAA TTG G | 430 | 1, 2 | Blast hits to 4 |
|  | 4-r | GCC CAC TCC TGT TAA AAT CCT ACC CGC ATT G |  |  |  |
|  |  |  |  |  |  |
| 5 | 5-f | ATA CCT ACA CAA CTT CTG ATT ATG CCT TTG TG | 362 | 1, 2 | Blast hits to 5 |
|  | 5-r | GCT CGA TAA ACA TAA TCA ATA TTT GAA AAA GTA TG |  |  |  |
|  |  |  |  |  |  |
| 6 | 6A/6B/6C/6D-f | AAT TTG TAT TTT ATT CAT GCC TAT ATC TGG | 250 | 1, 2 | Blast hits to group 6 |
|  | 6A/6B/6C/6D-r | TTA GCG GAG ATA ATT TAA AAT GAT GAC TA |  |  |  |
|  |  |  |  |  |  |
| 7B/C, 40 | 7C/7B/40-f | CTA TCT CAG TCA TCT ATT GTT AAA GTT TAC GAC GGG A | 260 | 1, 2 | Blast hits 7B/C and 40 |
|  | 7C/7B/40-r | GAA CAT AGA TGT TGA GAC ATC TTT TGT AAT TTC |  |  |  |
|  |  |  |  |  |  |
| 7F/A | 7F-f | CCT ACG GGA GGA TAT AAA ATT ATT TTT GAG | 826 | 1, 2 | Blast hits to 7F and 7A |
|  | 7F-r | CAA ATA CAC CAC TAT AGG CTG TTG AGA CTA AC |  |  |  |
|  |  |  |  |  |  |
| 8 | 8-f | GAT GCC ATG AAT CAA GCA GTG GCT ATA AAT C | 294 | 1, 2 | Blast hits to 8 |
|  | 8-r | ATC CTC GTG TAT AAT TTC AGG TAT GCC ACC |  |  |  |
|  |  |  |  |  |  |
| 9N/L | 9N/9L-f | GAA CTG AAT AAG TCA GAT TTA ATC AGC | 516 | 1, 4 | Blast hits to 9N and 9L |
|  | 9N/9L-r | ACC AAG ATC TGA CGG GCT AAT CAA T |  |  |  |
|  |  |  |  |  |  |
| 9V/A | 9V-f | CTT CGT TAG TTA AAA TTC TAA ATT TTT CTA AG | 753 | 1, 2 | Blast hits to 9V and 9A |
|  | 9V-r | GTC CCA ATA CCA GTC CTT GCA ACA CAA G |  |  |  |
|  |  |  |  |  |  |
| 9V/A | 9V/9A-f | GGG TTC AAA G TC AGA CAG TG A ATC TTA A | 816 | 1, 5 | Blast hits to 9V and 9A |
|  | 9V/9A-r | CCA TGA ATG A AA TCA ACA TT G TCA GTA GC |  |  |  |
|  |  |  |  |  |  |
| 10A | 10A-f | GGT GTA GAT TTA CCA TTA GTG TCG GCA GAC | 628 | 1, 2 | Blast hits to 10A |
|  | 10A-r | GAA TTT CTT CTT TAA GAT TCG GAT ATT TCT C |  |  |  |
|  |  |  |  |  |  |
| 10F/C, 33C | 10F/10C/33C-f | GGA GTT TAT CGG TAG TGC TCA TTT TAG CA | 248 | 1, 5 | Blast hits to 10F/C and 33C (or new33E) |
|  | 10F/10C/33C-r | CTA ACA AAT TCG CAA CAC GAG GCA ACA |  |  |  |
|  |  |  |  |  |  |
| 11 | 11A/11D-f | GGA CAT GTT CAG GTG ATT TCC CAA TAT AGT G | 463 | 1, 2 | Blast hits to new 11E, and 11D, 11A, 11F |
|  | 11A/11D-r | GAT TAT GAG TGT AAT TTA TTC CAA CTT CTC CC |  |  |  |
|  |  |  |  |  |  |
| 12A/B/F, 44, 46 | 12A/F-F | ACT CTT CCA AAT TCT TAT GCT TTT ATT GAT TC | 656 | 3 | Blast hits to 12A/B/F, 44, 46 |
|  | 12A/F-R | ATG AAT GAG AAA AGG AAC TTA AAA TTC ATA GC |  |  |  |
|  |  |  |  |  |  |
| 13 | 13-f | TAC TAA GGT AAT CTC TGG AAA TCG AAA GG | 655 | 1, 5 | Blast hits to 13 and new serotype (subtype |
|  | 13-r | CTC ATG CAT TTT ATT AAC CG C TTT TTG TTC |  |  | of 20 with 1 mismatch in F); R to 13 |
|  |  |  |  |  |  |
| 14 | 14-f | CTT GGC GCA GGT GTC AGA ATT CCC TCT AC | 208 | 1, 2 | Blast hits to 14; can cross-react with 15B/C |
|  | 14-r | GCC AAA ATA CTG ACA AAG CTA GAA TAT AGC C |  |  |  |
|  |  |  |  |  |  |
| 15A/F | 15A/15F-f | ATT AGT ACA GCT GCT GGA ATA TCT CTT C | 434 | 1, 2 | Blast hits to 15A, 15F |
|  | 15A/15F-r | GAT CTA GTG AAC GTA CTA TTC CAA AC |  |  |  |
|  |  |  |  |  |  |
| 15B/C | 15B/15C-F | AGG AAT CAG ATA TTA TCA TTA CTC ATG GTG | 496 | 3 | Can cross react with 14 |
|  | 15B/15C-R | TCA TGA CCC ATA GAA CTA TAT AAA AAG ACG |  |  |  |
|  |  |  |  |  |  |
| 16F | 16F-f | GAA TTT TTC AGG CGT GGG TGT TAA AAG | 717 | 1, 5 | Blast hits to 16F |
|  | 16F-r | CAG CAT ATA GCA CCG CTA AGC AAA TA |  |  |  |
|  |  |  |  |  |  |
| 17F | 17F-f | TTC GTG ATG ATA ATT CCA ATG ATC AAA CAA GAG | 693 | 1, 2 | Blast hits to 17F |
|  | 17F-r | GAT GTA ACA AAT TTG TAG CGA CTA AGG TCT GC |  |  |  |
|  |  |  |  |  |  |
| 18 | 18C/18F/18B/18A-f | CTT AAT AGC TCT CAT TAT TCT TTT TTT AAG CC | 573 | 1, 2 | Blast hits to 18 |
|  | 18C/18F/18B/18A-r | TTA TCT GTA AAC CAT ATC AGC ATC TGA AAC |  |  |  |
|  |  |  |  |  |  |
| 19A | 19A-f | GTT AGT CCT GTT TTA GAT TTA TTT GGT GAT GT | 478 | 1, 2 | Blast hits to 19A |
|  | 19A-r | GAG CAG TCA ATA AGA TGA GAC GAT AGT TAG |  |  |  |
|  |  |  |  |  |  |
| 19F | 19F-f | GTT AAG ATT GCT GAT CGA TTA ATT GAT ATC C | 304 | 1, 2 | Blast hits to 19F |
|  | 19F-r | GTA ATA TGT CTT TAG GGC GTT TAT GGC GAT AG |  |  |  |
|  |  |  |  |  |  |
| 20 | 20-f | GAG CAA GAG TTT TTC ACC TGA CAG CGA GAA G | 514 | 1, 2 | Blast hits to 20, possible new serotype |
|  | 20-r | CTA AAT TCC TGT AAT TTA GCT AAA ACT CTT ATC |  |  | within serogroup 20 |
|  |  |  |  |  |  |
| 21 | 21-f | CTA TGG TTA TTT CAA CTC AAT CGT CAC C | 192 | 1, 5 | Blast hits to 21 |
|  | 21-r | GGC AAA CTC AGA CAT AGT ATA GCA TAG |  |  |  |
|  |  |  |  |  |  |
| 22F/A | 22F/22A-f | GAG TAT AGC CAG ATT ATG GCA GTT TTA TTG TC | 643 | 1, 2 | Blast hits to 22F/A |
|  | 22F/22A-r | CTC CAG CAC TTG CGC TGG AAA CAA CAG ACA AC |  |  |  |
|  |  |  |  |  |  |
| 23A | 23A-f | TAT TCT AGC AAG TGA CGA AGA TGC G | 722 | 1, 5 | Blast hits to 23A |
|  | 23A-r | CCA ACA TGC TTA AAA ACG CTG CTT TAC |  |  |  |
|  |  |  |  |  |  |
| 23B | 23B-F | TTG TTA GTG GTA TTA AAT TGG GGA CTA CTA GG | 216 | 3 | Blast hits to 23B |
|  | 23B-R | ATA CCT ATC TGA AGT GTT ATT AAC CCA CCA AC |  |  |  |
|  |  |  |  |  |  |
| 23F | 23F-f | GTA ACA GTT GCT GTA GAG GGA ATT GGC TTT TC | 384 | 1, 2 | Blast hits to 23F |
|  | 23F-r | CAC AAC ACC TAA CAC TCG ATG GCT ATA TGA TTC |  |  |  |
|  |  |  |  |  |  |
| 24A/F | 24A/F-F | TCT CAA CCA AGA TAC AGA TTT TGA TTT TAC TC | 686 | 3 | Blast hits to 24A/F |
|  | 24A/F-R | TAT AAA CCT TTA GTA AAC ACT CTG CTT GAT CG |  |  |  |
|  |  |  |  |  |  |
| 29 | 29F | ATT ATC TCG GAT CAA ACA ATT CTT TTG TAA AC | 259 | 3 | Blast hits to 29 |
|  | 29R | AAC GCT AAC ATT AAA ACT AGA ACG AGT AAA CC |  |  |  |
|  |  |  |  |  |  |
| 31 | 31-f | GGA AGT TTT CAA GGA TAT GAT AGT GGT GGT GC | 701 | 1, 2 | Blast hits to 31 |
|  | 31-r | CCG AAT AAT ATA TTC AAT ATA TTC CTA CTC |  |  |  |
|  |  |  |  |  |  |
| 33F/A, 37 | 33F/33A/37-f | GAA GGC AAT CAA TGT GAT TGT GTC GCG | 338 | 1, 2 | Blast hits to 33F/A, 37 |
|  | 33F/33A/37-r | CTT CAA AAT GAA GAT TAT AGT ACC CTT CTA C |  |  |  |
|  |  |  |  |  |  |
| 34 | 34-f | GCT TTT GTA AGA GGA GAT TAT TTT CAC CCA AC | 408 | 1, 2 | Blast hits to 34 |
|  | 34-r | CAA TCC GAC TAA GTC TTC AGT AAA AAA CTT TAC |  |  |  |
|  |  |  |  |  |  |
| 35A/C, 42 | 35A/35C/42-f | ATT ACG ACT CCT TAT GTG ACG CGC ATA | 280 | 1, 5 | Blast hits to 35A/C, 42 |
|  | 35A/35C/42-r | CCA ATC CCA AGA TAT ATG CAA CTA GGT T |  |  |  |
|  |  |  |  |  |  |
| 35B | 35B-f | GAT AAG TCT GTT GTG GAG ACT TAA AAA GAA TG | 677 | 1, 2 | Blast hits to 35B |
|  | 35B-r | CTT TCC AGA TAA TTA CAG GTA TTC CTG AAG CAA G |  |  |  |
|  |  |  |  |  |  |
| 35F, 47F | 35F/47F-f | GAA CAT AGT CGC TAT TGT ATT TTA TTT AAA GCA A | 517 | 1, 2 | Blast hits to 35F, 47F |
|  | 35F/47F-r | GAC TAG GAG CAT TAT TCC TAG AGC GAG TAA ACC |  |  |  |
|  |  |  |  |  |  |
| 38, 25F/A | 38/25F/25A-f | CGT TCT TTT ATC TCA CTG TAT AGT ATC TTT ATG | 574 | 1, 2 | Blast hits to 38, 25F/A |
|  | 38/25F/25A-r | ATG TTT GAA TTA AAG CTA ACG TAA CAA TCC |  |  |  |
|  |  |  |  |  |  |
| 39 | 39-f | TCA TTG TAT TAA CCC TAT GCT TTA TTG GTG | 98 | 1, 5 | Blast hits to 39 |
|  | 39-r | GAG TAT CTC CAT TGT ATT GAA ATC TAC CAA |  |  |  |
|  |  |  |  |  |  |
| 45 | 45-f | GTT TAA TGG CTG ATG AAG TTA TTA TTG TTG | 238 | 3 | Blast hits to 45 |
|  | 45-r | TTT ACC ATC AGT GAA ATT TTA TCT TTG TTC |  |  |  |
|  |  |  |  |  |  |
| *cpsA* | *cpsA*-f | GCA GTA CAG CAG TTT GTT GGA CTG ACC | 160 | 1, 2 | Used in every PCR reaction |
|  | *cpsA*-r | GAA TAT TTT CAT TAT CAG TCC CAG TC |  |  |  |

References:

1. CDC Streptococcus Laboratory (accessed 28 Oct 2013): <http://www.cdc.gov/ncidod/biotech/strep/pcr.htm>
2. Pai R, Gertz RE, Beall B. (2006) Sequential multiplex PCR approach for determining capsular serotypes of *Streptococcus pneumoniae* isolates. J Clin Microbiol 44:124-31.
3. Saha SK, Darmstadt GL, Baqui AH, Hossain B, Islam M, Foster D, Al-Emran H, Naheed A, Arifeen SE, Luby SP, Santosham M, Crook D. (2008) Identification of serotype in culture negative pneumococcal meningitis using sequential multiplex PCR: implication for surveillance and vaccine design. PLoS One 3:e3576.
4. Dias CA, Teixeira LM, Carvalho Mda G, Beall B. (2007) Sequential multiplex PCR for determining capsular serotypes of pneumococci recovered from Brazilian children. J Med Microbiol 56:1185-8.
5. da Gloria Carvalho M, Pimenta FC, Jackson D, Roundtree A, Ahmad Y, Millar EV, O'Brien KL, Whitney CG, Cohen AL, Beall BW. (2010) Revisiting pneumococcal carriage by use of broth enrichment and PCR techniques for enhanced detection of carriage and serotypes. J Clin Microbiol 48:1611-8.
