# Supplementary material for: Streptococcus pneumoniae Carriage Prevalence in Nepal: Evaluation of a Method for Delayed Transport of Samples from Remote Regions and Implications for Vaccine Implementation
Source: PLoS One. 2014 Jun 6;9(6):e98739. doi: 10.1371/journal.pone.0098739 (PMC4048273; doi:10.1371/journal.pone.0098739)
Supplement: Table S2 — Primers Used for Multilocus Sequence Typing. (DOCX) [file pone.0098739.s002.docx]

| **Table S2. Primers Used for Multilocus Sequence Typing** | | | | |
| --- | --- | --- | --- | --- |
|  | | | | |
|  |  |  |  |  |
| **Locus** | **Primer** | **Direction** | **Sequence (5’ – 3’)** | **Reference** |
|  |  |  |  |  |
|  |  |  |  |  |
| *aroE* | aroE_csfF | F | CGTTTAGCTGCAGTTGTTGC | 1 |
|  | aroE_csfR | R | CCCACACTGGTGGCATTAAC | 1 |
|  |  |  |  |  |
| *gdh* | gdh_F | F | GGACAAACCAGCNAGYTT | 2 |
|  | gdh_R | R | GCTTGAGGTCCCATRCTNCC | 2 |
|  | Nepal_gdhF | F | CTACAACTTCTTTCGCTCCT | --^a^ |
|  | Nepal_gdhR | R | GGTGCCAAATGGACTTGGCA | --^a^ |
|  |  |  |  |  |
| *gki* | gki_F | F | GGCATTGGAATGGGATCACC | 2 |
|  | gki_R | R | TCTCCCGCAGCTGACAC | 2 |
|  |  |  |  |  |
| *recP* | recP_F | F | GCCAACTCAGGTCATCCAGG | 2 |
|  | recP_extR | R | AGATGGCTTGCCTGAAGC | --^b^ |
|  |  |  |  |  |
| *spi* | spi_extF | F | CGCTTAGAAAGGTAAGTTATGA | --^b^ |
|  | spi_R | R | GTGATTGGCCAGAAGCGGAA | 2 |
|  |  |  |  |  |
| *xpt* | xpt_csfF | F | CCACTACAACGGGAAATATTTGA | 1 |
|  | xpt_extR | R | CTTGAGTTTGCATTAGAGATCTGC | --^b^ |
|  |  |  |  |  |
| *ddl* | ddl_extF | F | AGCGTGTTCTGGAATCTGC | --^b^ |
|  | ddl_extR | R | AGGTCAACCAAACGCTCG | --^b^ |

1. The standard *gdh* MLST primers did not produce a PCR amplicon for some isolates; therefore, alternative *gdh* primers were designed for this study.
2. Redesigned original MLST primer to accommodate newer generation sequencing machines.

References:

1. Enright MC, Knox K, Griffiths D, Crook DW, Spratt BG. (2000) Molecular typing of bacteria directly from cerebrospinal fluid. Eur J Clin Microbiol Infect Dis 19: 627-630.
2. Enright MC, Spratt BG. (1998) A multilocus sequence typing scheme for *Streptococcus pneumoniae*: identification of clones associated with serious invasive disease. Microbiology 144: 3049-3060.
