# Supplementary material for: Streptococcus pneumoniae Carriage Prevalence in Nepal: Evaluation of a Method for Delayed Transport of Samples from Remote Regions and Implications for Vaccine Implementation
Source: PLoS One. 2014 Jun 6;9(6):e98739. doi: 10.1371/journal.pone.0098739 (PMC4048273; doi:10.1371/journal.pone.0098739)
Supplement: Table S3 — Results of serogroup/type retesting by Quellung. (DOCX) [file pone.0098739.s003.docx]

| **Table S3. Results of serogroup/type retesting by Quellung.** | | |
| --- | --- | --- |
|  |  |  |
|  |  |  |
| **Original PCR designation** | **Quellung result** | **Final designation** |
|  |  |  |
|  |  |  |
| Nontypeable (NT; n = 66) | NT (n = 58) | NT |
|  | Stock culture nonviable (n = 1) | NT |
|  | 15B (n = 1) | 15B/C^a^ |
|  | 19F (n = 1) | 19F |
|  | 15A (n = 1) | 15A/F^a^ |
|  | 35F (n = 1) | 35F, 47F^a^ |
|  | 29 (n = 1) | 29 |
|  | 10A (n = 1) | 10A |
|  | 28F (n = 1) | 28F |
|  |  |  |
| Serotype 15B/C (n = 54) | 15B/C (n = 48) | 15B/C |
|  | Stock culture nonviable (n = 1) | 15B/C |
|  | 14 (n = 5) | 14 |
|  |  |  |
| Serotype 14 (n = 40) | 14 (n = 22) | 14 |
|  | Stock culture nonviable (n = 1) | 14 |
|  | NT (n = 11) | NT^b^ |
|  | 7B (n = 2) | 7B/C, 40^a^ |
|  | 7C (n = 1) | 7B/C, 40^a^ |
|  | 29 (n = 1) | 29 |
|  | 16F (n = 1) | 16F |
|  | 15C (n = 1) | 15B/C^a^ |
|  |  |  |

a. Note that the final designation is given as indicated, to match the PCR designations.

b. We presumed the PCR result was correct but that the *cps* locus is no longer functional: the MLST genotyping indicated that 5 isolates were in CC63 and 1 isolate was in CC7645/7646 and all other isolates in those two CCs were serotype 14. A further four isolates were CC Singletons and 1 isolate was not genotyped so it was difficult to infer much more from those. In total, all 11 serotype designations were changed from 14 to NT in the master database and all downstream analyses.
